# Molecular basis for sensitivity and acquired resistance to gefitinib in HER2-overexpressing human gastric cancer cell lines derived from liver metastasis

**DOI:** 10.1038/sj.bjc.6603459

**Published:** 2006-11-07

**Authors:** H Yokoyama, Y Ikehara, Y Kodera, S Ikehara, Y Yatabe, Y Mochizuki, M Koike, M Fujiwara, A Nakao, M Tatematsu, H Nakanishi

**Affiliations:** 1 Division of Oncological Pathology, Aichi Cancer Center Research Institute, 1-1 Kanokoden, Chikusa-ku, Nagoya 464-8681, Japan; 2 Department of Surgery II, Nagoya University School of Medicine, 65 Tsurumai-cho, Shouwa-ku, Nagoya 466-8550, Japan; 3 Pathology and Molecular Diagnostics, Aichi Cancer Center Hospital, Chikusa-ku, Nagoya 464-8681, Japan; 4 Department of Gastroenterological Surgery, Aichi Cancer Center Hospital, 1-1 Kanokoden, Chikusa-ku, Nagoya 464-8681, Japan

**Keywords:** gastric cancer, liver metastasis, gefitinib, trastuzumab, HER2, PI3K/Akt, drug resistance

## Abstract

Gastric cancer metastasised to the liver was found to overexpress HER2 at a significantly higher incidence than primary gastric cancers. The purpose of the present study was to investigate the possibility of molecular therapy targeting HER2 overexpression in gastric cancer liver metastasis. We developed three new HER2-overexpressing gastric cancer cell lines (GLM-1, GLM-2, GLM-4) without epidermal growth factor receptor (EGFR) mutations derived from such liver metastasis, two of which had HER2 gene amplifications. All these GLM series of cell lines were highly sensitive to gefitinib *in vitro*, a specific inhibitor of EGFR tyrosine kinase (Iressa) rather than anti-HER2 antibody trastuzumab (Herceptin), whereas most of the HER2 low-expressing counterparts were not. In these HER2-overexpressing GLM series, protein kinase B (Akt), but not extracellular signal-regulated kinase 1/2 (ERK1/2), was constitutively phosphorylated, and gefitinib efficiently inhibited this Akt phosphorylation, induced strong apoptosis *in vitro* and exhibited antitumour activity in tumour xenografts in nude mice. This gefitinib-mediated antitumour effect in xenograft was significantly potentiated by trastuzumab treatment. On the other hand, gefitinib-resistant cells (GLM-1R) exhibited increased EGFR expression, followed by constitutive activation of mitogen-activated protein kinase (MAPK) pathway. These results suggest that the antitumour effect of gefitinib is due to the effective inhibition of HER2-driven constitutive activation of phosphatidylinositol-3-kinase (PI3K)/Akt pathway, and that the acquired resistance to gefitinib is due to the constitutive activation of Ras/MAPK pathway in compensation for PI3K/Akt pathway. Gastric cancer liver metastasis with HER2 overexpression would be a potential molecular target for gefitinib and trastuzumab.

Although the survival of patients with gastric cancer has improved, gastric cancer still remains one of the major causes of cancer death in Japan as well as in some Western countries. Liver metastasis is an important prognostic factor, accounting for 10–15% of gastric cancer recurrences after curative resection (Marrelli *et al*, 2004). This is a noncurable, fatal disease with a 5-year survival of less than 10% (Sakamoto *et al*, 2003). To date, however, the molecular basis of its growth and metastasis remains essentially unknown, and there are no effective therapeutic modalities including molecular targeting therapy.

HER family is a type I receptor tyrosine kinase including HER1 (epidermal growth factor receptor (EGFR)), HER2 (*neu*/*erbB*2), HER3 (*erb*B3) and HER4 (*erb*B4) that regulates essential cellular functions, such as growth, differentiation and apoptosis in normal tissues, and their activation is associated with carcinogenesis and progression in solid tumours (Yarden, 2001). The binding of ligands to the extracellular region of HER family induces receptor homo- or heterodimerisation and activation of cytoplasmic tyrosine kinase (TK), which in turn leads to autophosphorylation and initiation of downstream signalling pathways (Muthuswamy *et al*, 1999). Among the members of HER family, HER2 is overexpressed in 10–30% of patients with gastric cancer and is known to lead to a poor patient outcome owing to malignant progression, for example, metastasis and resistance to chemotherapy such as that found in breast cancer (David *et al*, 1992; Uchino *et al*, 1993). A unique feature of HER2, as revealed by recent crystallo-structural analysis of HER2 protein, is that HER2 has a fixed conformation that resembles a ligand-activated state and can interact with other HER receptors to form EGFR/HER2 and HER2/HER3 (HER4) heterodimers even in the absence of a cognate ligand, unlike EGFR and HER3 (HER4) (Cho *et al*, 2003). Therefore, HER2 is capable of constitutively transducing signals downstream to such as the Ras/mitogen-activated protein kinase (MAPK) and phosphatidylinositol-3-kinase (PI3K)/protein kinase B (Akt) pathways by upregulation of HER2 in addition to the activation by mutation or amplification in the absence of ligand binding. Consequently, HER2 overexpression mediates multiple pathological responses including a chemokine receptor, CXCR4-associated metastasis (Li *et al*, 2004) and a multidrug resistance owing to activation of PI3K/Akt pathway (Knuefermann *et al*, 2003). These findings suggest that HER2-overexpressing gastric cancers are potential targets for antitumour molecular therapy.

Gefitinib, an orally active, quinazoline tyrosine kinase inhibitor selective for EGFR, has been proved to have a clinically significant antitumour activity in lung cancers, and is now approved for use in several countries, including Japan and the USA. Recent studies demonstrated that somatic mutations within the ATP-binding pocket of the tyrosine kinase domain of the EGFR, including small in-frame deletions and missense substitutions, are present in a subset of lung adenocarcinomas and confer susceptibility to the gefitinib in lung cancer patients (Lynch *et al*, 2004; Paez *et al*, 2004). However, EGFR mutation was found to be generally rare in most malignancies except lung cancers, and gefitinib-sensitive cancers without EGFR mutations have been reported in HER2-overexpressing breast cancer cell lines as representative examples (Moasser *et al*, 2001; Moulder *et al*, 2001). To date, however, the sensitivity of HER2-overexpressing gastric cancers to gefitinib remains essentially unknown.

Recently, we found that the HER2 positivity rate was significantly higher in gastric cancer liver metastasis than in primary tumours. Based on this clinical observation and the experimental evidences that some HER2-overexpressing breast cancer cell lines are sensitive to gefitinib, we speculated that HER2-overexpressing gastric cancer liver metastases were susceptible to gefitinib. To test this possibility, we established three new HER2-overexpressing gastric cancer cell lines (GLM-1, GLM-2 and GLM-4) derived from liver metastasis (Nakanishi *et al*, 2005) and examined the sensitivity of these cell lines to gefitinib. As expected, all the HER2-overexpressing gastric cancer cell lines exhibited high sensitivity to gefitinib both *in vitro* and *in vivo*. Here we report on the mechanism underlying the sensitivity and acquired resistance of these cell lines to gefitinib, and discuss possible molecular targeting therapy against patients with HER2-overexpressing gastric cancer liver metastasis.

## MATERIALS AND METHODS

### Compounds

Gefitinib (ZD1839; Iressa) was provided by AstraZeneca (Macclesfield, UK). Trastuzumab (Herceptin, Genentech, CA, USA), a humanised anti-HER2 monoclonal antibody, was purchased from Chugai Pharmaceuticals (Tokyo, Japan). Human recombinant EGF and heregulin were obtained from R&D Systems (Mineapolis, MN, USA). Phosphatidylinositol-3-kinase (PI3K) inhibitor (LY294002) and mitogen-activated protein/extracellular signal-regulated kinase kinase (MEK) 1/2 inhibitor (U0126) were obtained from Cell Signaling Technology (Beverly, MA, USA). Antibodies used were as follows: for Western blotting analysis, mouse monoclonal antibodies to total Erk1/2 and phospho-Erk1/2 (Thr202/Tyr204), rabbit polyclonal antibodies to total Akt, phospho-Akt (Ser473) and to PTEN (phosphatase and tensin homologue gene) (Cell Signaling Technology, Beverly, MA, USA); mouse monoclonal antibody to HER2 (Cell Signaling Technology), HER3 (clone 2F12, Upstate, Lake Placid, NY, USA), and *α*-tubulin (clone DM1A, Sigma-Aldrich); rabbit polyclonal antibody to CXCR4 (Abcam Ltd, Cambridge, UK). For immunohistochemistry, mouse monoclonal antibody to EGFR (Novocastra Labs, Newcastle, UK), rabbit polyclonal antibody to HER2 (DAKO Cytomation, Glostrup, Denmark) and HER3 (Ab-10, NeoMarkers, Fremont, CA, USA) and rabbit monoclonal antibody to phospho-Akt (Ser473) (736E11, Cell Signaling Technology) were used. For flow cytometry, mouse monoclonal antibody to EGFR (NeoMarkers) was used.

### Tissue samples

Sixty-four primary stomach tumours from stage II–III gastric cancer patients who underwent gastrectomy between 1995 and 1996, and 20 metastatic liver tumours from stage IV gastric cancer patients who underwent partial hepatectomy (17 patients), biopsy (two patients) and autopsy (one patient) during 1991 and 2005 in the Department of Gastroenterological Surgery, Aichi Cancer Center Hospital were used in this study, which was approved by the Institutional Ethical Committee of the Aichi Cancer Center.

### Cell lines

GLM-1 and GLM-2 cell lines were recently established in our laboratory from liver metastasis of Japanese gastric cancer patients who were surgically resected in Aichi Cancer Center Hospital as reported previously (Nakanishi *et al*, 2005). GLM-4 is a newly established cell line in our laboratory from liver metastases obtained from a 65-year-old Japanese man suffering from stage IV gastric cancer with multiple liver metastasis at autopsy. These tumour tissues were obtained from patients or their family with written informed consent. Establishment of each cell line was performed according to the method described previously (Nakanishi *et al*, 1995). Other human gastric cancer cell lines derived from liver metastases from a Caucasian gastric cancer patient (NCI-N87) were obtained from ATCC (Manassas, VA, USA). The conventional human gastric cancer cell lines, GCIY, MKN-28, MKN-45 and MKN-74 were obtained from RIKEN Cell Bank (Tsukuba, Japan). HSC-43 cell line was kindly provided by Dr K Yanagihara (National Cancer Center Research Institute, Tokyo, Japan). These cell lines were maintained in DMEM (Nissui Pharmaceutical Co., Ltd, Tokyo, Japan) or RPMI1640 (Sigma, St Louis, MO, USA) supplemented with 10% fetal bovine serum (FBS) (GIBCO, Grand Island, NY, USA), 100 U ml^−1^ penicillin and 100 *μ*g ml^−1^ streptomycin in plastic dishes (Falcon, Franklin Lakes, NJ, USA), and incubated at 37°C in 5% CO_2_. GLM-2 and GLM-4 lines were cultured in type I collagen-coated dishes (IWAKI, Asahi Techno Glass, Japan). A subclone of GLM-1 cells resistant to gefitinib (GLM-1R) was isolated after four cycles of a selection treatment consisting of continuous exposure of parental cells to 1 *μ*M gefitinib for 1–2 weeks, followed by permitting regrowth of surviving cells and subsequent passage *in vitro* for each.

### *In vitro* cell growth assay

Cells were harvested with trypsin/EDTA, plated at 5 × 10^4^ cells per 24-well plastic plate or type I collagen-coated plate (for GLM-2 and GLM-4 cells) in Dulbecco's modified Eagle's medium (DMEM) supplemented with 10% FBS, and then treated with increasing doses of gefitinib (0.01, 0.1, 1 and 10 *μ*M) or trastuzumab (0, 1, 10, and 100 *μ*g ml^−1^) on days 1 and 3. The number of viable cells was counted on day 4 with a hemocytometer in triplicate.

### Cell cycle analysis by flow cytometry

To determine the percentage of cells at various phases of cell cycle, exponentially growing cells were treated with gefitinib at a concentration of 10 *μ*M for 24 h, untreated control and gefitinib-treated cells (1 × 10^6^) were analysed for nuclear DNA after propidium iodide staining using CycleTEST Plus Kit (Becton Dickinson, San Jose, CA, USA) according to the manufacturer's instructions. Flow cytometric analysis was performed in FACSCalibur (Becton Dickinson). Data collected from 10 000 cells for each experiment were analysed by ModFit software (Verity Software House, Topsham, ME, USA).

### Apoptosis assay

Apoptosis induced by gefitinib was quantitated by measuring caspase-3/7 activity using Apo-one™ Homogeneous Caspase-3/7 Assay Kit (Promega Corp., Madison, WI, USA). Cells were harvested with trypsin/EDTA and were plated at 1 × 10^4^ cells per 96-well plastic plate or type I collagen-coated plate (for GLM-2 and GLM-4 lines) in DMEM and treated with increasing doses of gefitinib (0, 0.1, 1 and 10 *μ*M), LY294002 (0, 1, 10 and 50 *μ*M) and U0126 (0, 1, 10 and 50 *μ*M) on day 1 in the presence of 10% FBS. After 24 h, caspase-3/7 activity of the cells was then measured by adding an aliquot of the Homogeneous Caspase-3/7 reagent to each well, shaking the plate for 1 h at room temperature, and measuring the fluorescence intensity by a fluorescent plate reader (Wallac Co., Turku, Finland). Inhibition of gefitinib-induced apoptosis by Caspase-3 tetrapeptide inhibitor, DMQD-CHO (Peptide Institute, Osaka, Japan) was examined by the pretreatment of cells plated in 96-well plates with increasing doses of DMQD-CHO (0, 1, 10 *μ*M) for 1 h, followed by the treatment with increasing doses of gefitinib (0, 0.1, 1 and 10 *μ*M).

### Western blot analysis

The monolayer culture cells were maintained on 60 mm dishes in medium supplemented with 10% FBS. To evaluate the effects of gefitinib under ligand-mediated stimulation, cells were incubated in serum-free medium for 24 h, and exposed to gefitinib at increasing concentrations (0, 0.1, 1 and 10 *μ*M) for 2 h at 37°C. Unless otherwise indicated, cancer cells were incubated for different time periods with either EGF or heregulin (10 or 50 ng ml^−1^). Cells were then lysed at 4°C in lysis buffer (10 mM Tris-HCl, pH 7.5, 150 mM NaCl, 1% Triton X-100, 1 mM EDTA and complete Protease Inhibitor Cocktail). Protein concentration was determined by Lowry assay (DC Protein Assay; Bio-Rad, Hercules, CA, USA), and 10 *μ*g cell aliquots were directly lysed in Laemmli sample buffer for subsequent immunoblotting with antibodies as described previously (Ikehara *et al*, 2004). In brief, whole cell lysates were separated by SDS–PAGE under reducing conditions, and transferred to polyvinylidene difluoride membranes (Amersham- Pharmacia Biotech, Buckinghamshire, UK) and immunoblotted with antibodies described above. Bound antibodies were visualised using Western Lightning Chemiluminescence Plus (PerkinElmer Life Sciences, Boston, MA, USA).

### Cell-based detection of phosphorylation of Akt and Erk by ELISA

Alteration of Akt and Erk1/2 phosphorylation by treatment with specific inhibitor was directly measured on 96-well cultured cells using a cell-based ELISA assay as described previously (Versteeg *et al*, 2000). Cells were seeded at 1 × 10^4^ cells per 96-well plastic plate on day 0 and allowed to grow in the presence of 10% FBS for 24 h. On day 1, the relevant cells were treated with inhibitors such as PI3K inhibitor (LY294002) and MEK 1/2 inhibitor (U0126) for 2 h, and then phospho-protein and total protein were quantitated by the Cellular Activation of Signaling ELISA (CASE Kit, SuperArray Bioscience, Frederick, MD, USA) following the manufacturer's instruction. Briefly, the treated cells were fixed in 4% formaldehyde in phosphate buffered saline (PBS), quenched with buffer containing H_2_O_2_ and NaN_3_, followed by blocking nonspecific reactions for 1 h. Primary antibody specific for both phospho-Akt (Ser473) and pan-Akt was then added to two identical sets of each well and incubated for 1 h at room temperature. After washing with PBS, the wells were incubated with HRP-conjugated secondary antibody for 1 h, and the colour was developed with 10 min incubation at room temperature and measured at 450 nm absorbance on a plate reader.

### Sequence analysis of EGFR and HER2 gene

Total RNAs from GLM-1 and GLM-2 cells were extracted using the RNAeasy kit (Qiagen, Valencia, CA, USA). Complementary DNAs were synthesised with oligo (dT) primers in a 20 *μ*l total volume reaction mixture using a superscript preamplification system (Invitrogen, Carlsbad, CA, USA). RT–PCR was performed with high-fidelity KOD polymerase pulse (Toyobo, Osaka, Japan) to amplify the first four exons (exons 18–21) of the seven exons (exons 18–24) that code for TK domain of the EGFR and HER2 gene. The PCR products were purified and subjected to the cycle-sequence with the Big Dye Terminator v3.1 cycle sequence kit (Applied Biosystems, Foster City, CA, USA).

### Immunohistochemical analysis

Subcutaneous tumours formed 1 month after injection of tumour cells into male nude mice of KSN strain were removed and fixed in 10% buffered formalin for 24 h. Formalin-fixed and paraffin-embedded sections (4 *μ*m) were used for immunohistochemistry and fluorescence *in situ* hybridisation (FISH). For antigen retrieval, the sections were treated with microwave at 98°C for 10 min. After blocking nonspecific reactions, the sections were incubated at 4°C overnight with antibodies to EGFR, HER2 and HER3 with optimal dilution. After washing with PBS, the sections were incubated with biotinylated secondary antibodies for 30 min. The sections were washed again with PBS, then incubated with streptavidin–peroxidase complex (Vectastain ABC kit, Vector Laboratories, Burlingame, CA, USA) for 60 min. The chromogen was developed with 0.01% diaminobenzidine (DAB), and the sections were counterstained with Meyer's hematoxylin. Immunohistochemistry for HER2 as described above is a conventional method with a three-step visualisation system which is similar to HercepTest (Dako Cytomation, Carpinteria, CA, USA) in terms of the use of the same polyclonal antibody and estimation system, but is different from HercepTest in the antigen retrieval method and visualisation method. EGFR and HER2 protein expression was scored for each of the 3+, 2+, 1+ and 0 categories based on the membrane staining according to the criteria of HercepTest. Tumours with staining scores of 2+ and 3+ were evaluated as positive. Histology was simply subdivided into the intestinal type and diffuse type according to Lauren's classification.

### FISH analysis

Amplification of the c-*erb*B-2 gene was determined by dual-colour FISH method using the Passvision HER-2 DNA probe Kit (Vysis Inc. Downers Grove, IL, USA) according to the manufacturer's protocol. The HER-2/*neu*-Spectrum Orange probe contains a DNA sequence specific for the c-*erb*B-2 human gene locus and hybridises to region 17q11.2–q12 of human chromosome. The CEP 17 (chromosome enumeration probe 17)/Spectrum Green probe used as a control contains alpha-satellite DNA that hybridises to the D17Z1 locus (centromere region of chromosome 17). The nucleus was counterstained with 4′,6-diamidino-2-phenylindole (DAPI). The slides were observed under BX60 fluorescence microscope equipped with digital camera (DP50) (Olympus, Tokyo, Japan), and the images were captured on a Windows PC with Viewfinder Lite software. A cell was considered to show amplification when a definite cluster or more than 10 signals for c-*erb*B-2 was found.

### Tumour xenograft studies

To examine antitumour activity of gefitinib *in vivo*, growing cells were harvested with trypsin–EDTA, washed with PBS, and 5 × 10^6^ cells in 0.2 ml PBS were injected subcutaneously into the left abdominal flanks of 8-week-old male nude mice of KSN strain (Shizuoka Laboratory Animal Center, Hamamatsu, Japan) maintained under specific-pathogen-free (SPF) conditions. Mice (*n*=5) were orally administered gefitinib with a gastric tube at a dose of 0, 100 or 150 mg per kg per day from day 10 after injection, five times per week for 3–6 weeks. In the control groups, mice were orally administered vehicle (0.5% Polysorbate, Merck, Damstadt, Germany) in the same manner. In the treatment with trastuzumab, mice were given intraperitoneal injection at a dose of 20 mg per kg per day, twice a week for 6 weeks. Tumour maximum diameter (*L*) and the right angle diameter to that axis (*W*) were measured every week. Tumour volume was estimated by the following formula, *L* × *W* × *W* × 1/2. All experiments were carried out with the approval of the Institutional Ethical Committee for Animal Experiment of Aichi Cancer Center Research Institute and met the standard as defined by the UKCCR guidelines (Workman *et al*, 1998).

### Statistical analysis

The statistical significance of the differences in data between each treatment groups was determined by applying Student's *t* test. Differences in the incidence between the groups were analysed with *χ*^2^ test or Fisher's exact test. A *P*-value <0.05 was considered significant.

## RESULTS

### Incidence of HER2 overexpression in primary and metastatic liver tumours

HER2 positivity rate (65%), as determined by immunohistochemistry, was significantly higher in the metastatic liver tumours than in the primary tumours of the stomach (38%) (*P*<0.05). Histologically, HER2 positivity rate was higher in intestinal type than diffuse type, particularly in intestinal-type liver metastatic lesions (90%). In contrast, EGFR and HER3 positivity rate did not differ between primary tumours and liver metastasis ranging from 25 to 30% in gastric cancer patients, indicating selective overexpression of HER2 in metastatic liver tumours.

### HER2 overexpression in human gastric cancer cell lines derived from liver metastasis

**Figure 1:**
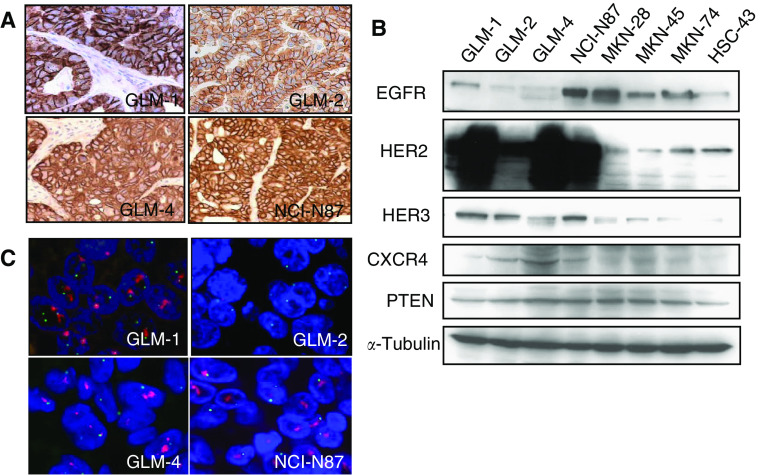
HER2 overexpression in human gastric cancer cell lines derived from liver metastasis. (**A**) HER2 expression determined by immunohistochemistry of xenografts in nude mice formed after subcutaneous injection of GLM-1, GLM-2, GLM-4 and NCI-N87 cells. Note the strong membranous staining in these cells. (**B**) Expression of EGFR, HER2, HER3, CXCR4 and PTEN as determined by Western blotting of cultured cell lysates. (**C**) Amplification of HER2 gene determined by dual-colour FISH of xenografts in nude mice. HER2 gene amplification in a cluster pattern (red) was observed in GLM-1, GLM-4 and NCI-N87 cells.

Immunohistochemistry of nude mouse xenografts showed that all the liver metastasis-derived intestinal-type gastric cancer cell lines (GLM-1, GLM-2, GLM-4 and NCI-N87) overexpressed HER2 on their cell surface, and were judged as positive (2+ or 3+) based on the criteria of HercepTest (Figure 1A). In contrast, HER2 expression of other gastric cancer cell lines including the diffuse-type (MKN-45 and HSC-43) and intestinal-type cell lines (MKN-28 and MKN-74) was low and judged as negative (0 or 1+). Moderate HER3 expression in the cytoplasm together with cell surface was also observed in GLM-1, GLM-4 and NCI-N87 cells. In contrast, EGFR overexpression was observed only in NCI-N87 cells (data not shown). Western blot analysis confirmed apparent overexpression of HER2 in GLM-1, GLM-2, GLM-4 and NCI-N87 cells along with EGFR expression in NCI-N87 cells, and HER3 expression in GLM-1, GLM-2 and NCI-N87 cells (Figure 1B). FISH analysis clearly demonstrated HER2 gene amplification in a cluster pattern in GLM-1, GLM-4 and NCI-N87 cells, but not GLM-2 cells (Figure 1C). Furthermore, expression of CXCR4, which is known to be upregulated by HER2 overexpression, was found to be slightly increased in HER2-overexpressing cancer cell lines (Figure 1B).

### Growth inhibition and apoptosis induction by gefitinib in HER2- overexpressing cells

**Figure 2:**
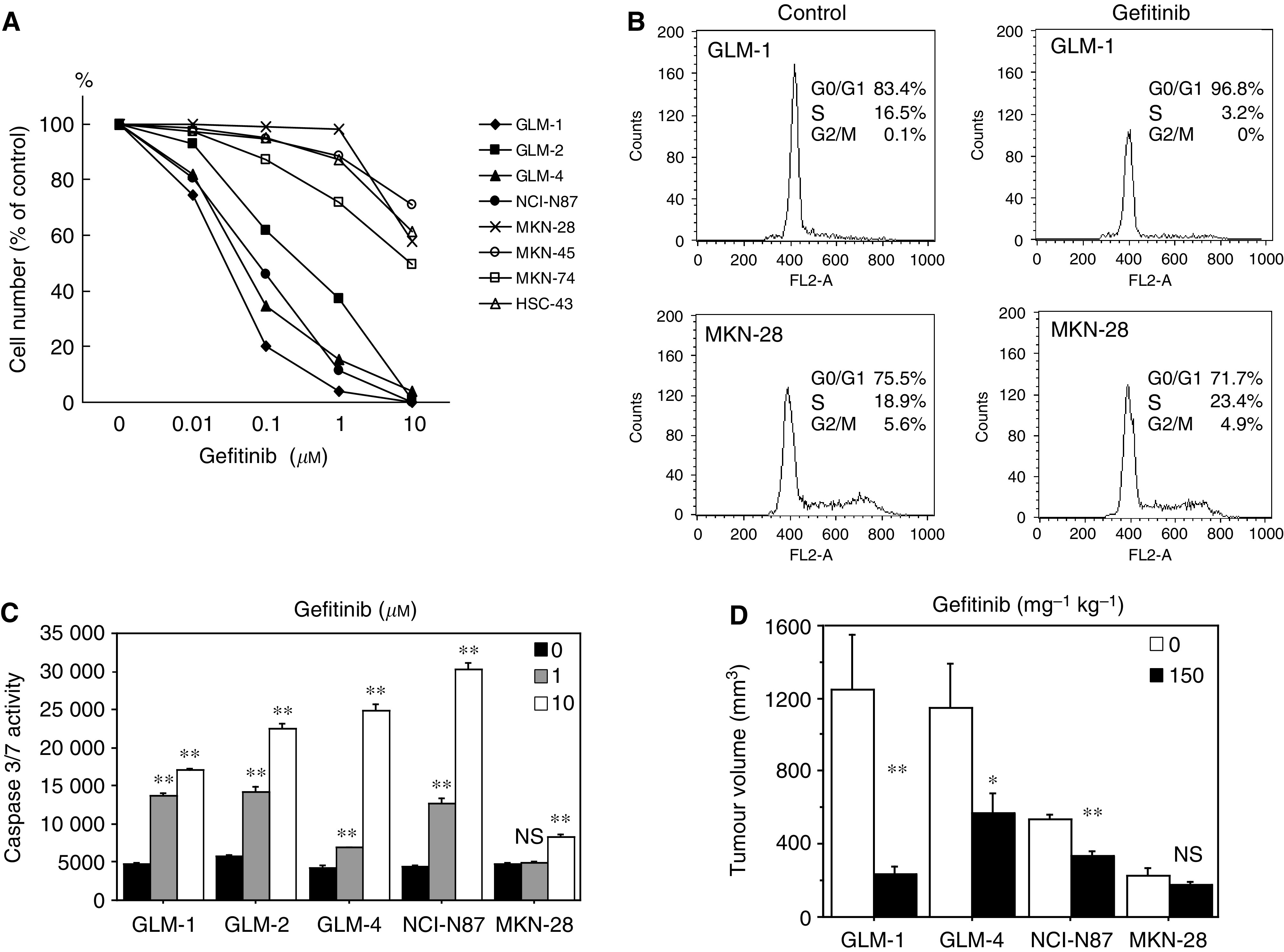
Growth inhibition and apoptosis induction by gefitinib in HER2-overexpressing cancer cell lines both *in vitro* and *in vivo*. (**A**) Growth inhibition by gefitinib *in vitro* in various human gastric cancer cell lines. Cells were plated in 24-well dish and treated twice with increasing doses of gefitinib, followed by cell counting after a 4-day culture. (**B**) Flow cytometric analysis of cell cycle of HER2-overexpressing and low-expressing cells with or without gefitinib treatment. DNA histogram and % of each cell cycle phase are shown. (**C**) Apoptosis induction by gefitinib in HER2-overexpressing and low-expressing cells at the increasing drug concentrations of 0 *μ*M (▪), 1.0 *μ*M (░) and 10 *μ*M (□), as evaluated by measuring caspase 3/7 activity. (**D**) Inhibitory effects of gefitinib on the growth of HER2-overexpressing and low-expressing tumour xenografts in nude mice. Tumour cells were injected subcutaneously into nude mice, and gefitinib was orally administered at a dose of 0 (□), 150 mg per kg per day (▪), five times per week for 3 weeks. Ns, not significant; ^*^P<0.01; ^**^P<0.001 (*vs* control). Bars=s.e.

The growth of HER2-overexpressing cell lines (GLM-1, GLM-2, GLM-4 and NCI-N87) *in vitro* was markedly inhibited by gefitinib with IC_50_ of 0.028, 0.32, 0.078 and 0.091 *μ*M, whereas most of the HER2 low-expressing gastric cancer cell lines (GCIY, MKN-28, MKN-45, MKN-74) were resistant to gefitinib with IC_50_ more than 10 *μ*M (Figure 2A), indicating a correlation between degree of HER2 expression and gefitinib sensitivity in gastric cancer cell lines. Cell cycle analysis by a flow cytometer showed that gefitinib treatment (10 *μ*M) resulted in the depletion of S phase cells from 16.5 to 3.2%, accumulation of G_0_–G_1_ phase cells (83.4–96.8%) and virtually no change in G_2_–M phase cells. This indicated the weak induction of G_1_ arrest of cell cycle in HER2-overexpressing GLM-1 cells by gefitinib. On the other hand, the proportion of each stage of the cell cycle in HER2 low-expressing MKN-28 cells changed from 18.9 to 23.4% in S phase cells, from 75.5 to 71.7% in G_0_-G_1_ phase cells, and from 5.6 to 4.9% in G_2_-M phase cells after gefitinib treatment. Thus, there was no indication of G_1_ arrest by gefitinib in HER2 low-expressing MKN-28 cells (Figure 2B). Furthermore, caspase-3/7 assay revealed that gefitinib induced apoptosis in HER2-overexpressing cancer cell lines in a dose- and time-dependent manner (Figure 2C). Caspase 3-dependent apoptosis induction by gefitinib was confirmed by the suppression of gefitinib-induced apoptosis with a caspase-3 inhibitor (DMQD-CHO) (data not shown).

The effect of gefitinib on the growth of tumour xenograft in nude mice was also examined. Daily oral administration of gefitinib (150 mg kg^−1^, five times a week) significantly suppressed tumour growth of HER2-overexpressing cells (GLM-1, GLM-4 and NCI-N87) in a dose-dependent manner, whereas no significant inhibition of tumour growth was observed with HER2 low-expressing cell lines (MKN-28) (Figure 2D). HER2-overexpressing GLM-2 cells, without gene amplification, have no tumorigenicity in nude mice.

### Effects of PI3K inhibitor and MEK inhibitor on the apoptosis induction

**Figure 3:**
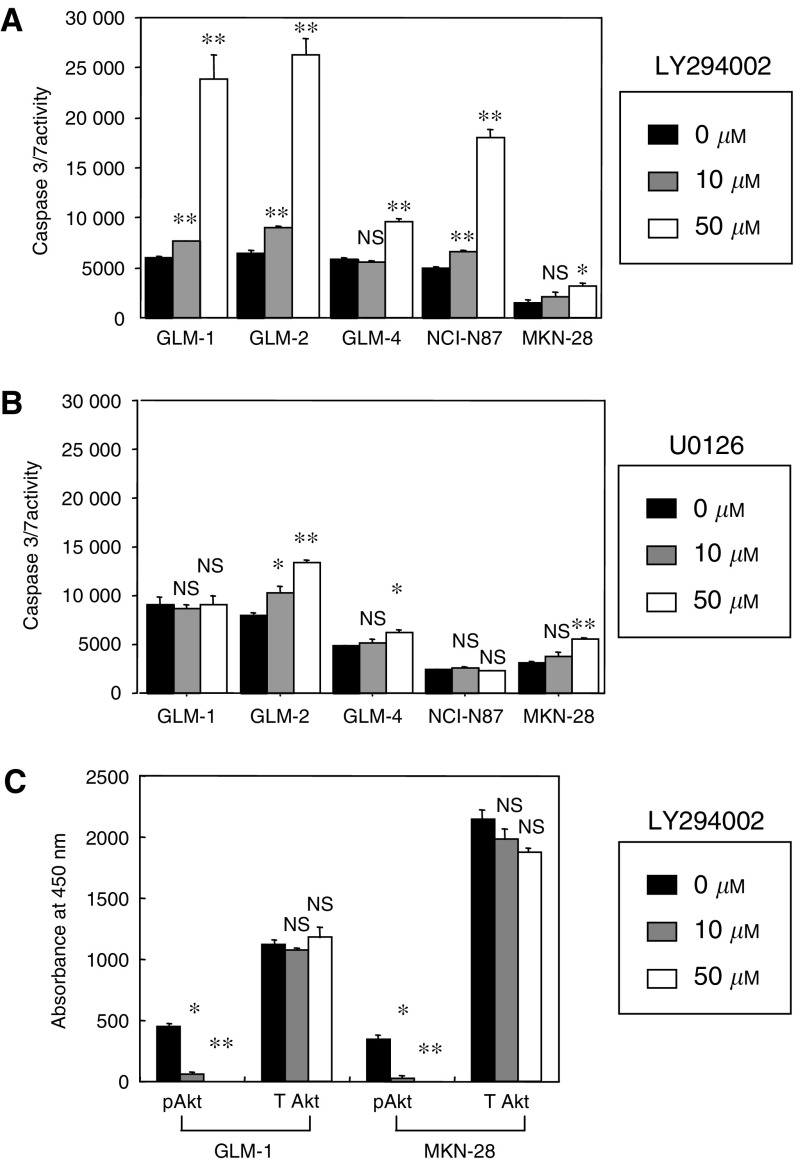
Effects of PI3K inhibitor (LY294002) and MEK inhibitor (U0126) on apoptosis induction and Akt phosphorylation of HER2-overexpressing and low-expressing cancer cell lines. (**A**) Apoptosis induction by LY294002. Cells were plated at 1 × 10^4^ cells per 96-well dish and LY294002 concentrations of 0 *μ*M (□), 10 *μ*M (░), 50 *μ*M (▪) were added 1 day after seeding and cultured for 24 h. Apoptosis was measured with caspase 3/7 assay. (**B**) Apoptosis induction by U0126 at concentrations of 0 *μ*M (□), 10 *μ*M (░), 50 *μ*M (▪). (**C**) Inhibition of Akt phosphorylation by LY294002. Cells were plated at 1 × 10^4^ cells per 96-well dish in the presence of 10% FBS and 1 day after seeding, LY294002 at respective concentrations of 0 *μ*M (□), 10 *μ*M (░), 50 *μ*M (▪) were added and cultured for 2 h. Phosphorylated Akt (pAkt) and total Akt (T Akt) were measured with AKT 473 ELISA Kit. NS, not significant; ^*^P<0.01; ^**^P<0.001 (*vs* control). Bars=s.e.

The basal apoptotic rate was significantly higher in HER2-overexpressing cell lines (GLM-1, GLM-2, GLM-4 and NCI-N87) than HER2 low-expressing cell line (MKN-28) in the absence of apoptosis-inducing stimuli (*P*<0.001) (Figure 3). Marked enhancement of apoptosis by a specific inhibitor for PI3K (LY294002) was observed in HER2-overexpressing cell lines (*P*<0.001), but not MKN-28 cell line (Figure 3A). MEK1/2 inhibitor (U0126) only partially enhanced apoptosis in GLM-2, GLM-4 and MKN-28 cells (Figure 3B). However, ELISA assay for protein-phosphorylation revealed that LY294002 significantly reduced phospho Akt, but not total Akt, in a dose-dependent manner in both HER2-overexpressing and HER2 low-expressing cells (Figure 3C), indicating that HER2-overexpressing cells depend for their survival on the PI3K/Akt pathway rather than the MAPK pathway as major downstream signalling pathways.

### Effects of gefitinib on EGFR downstream signalling pathways

**Figure 4:**
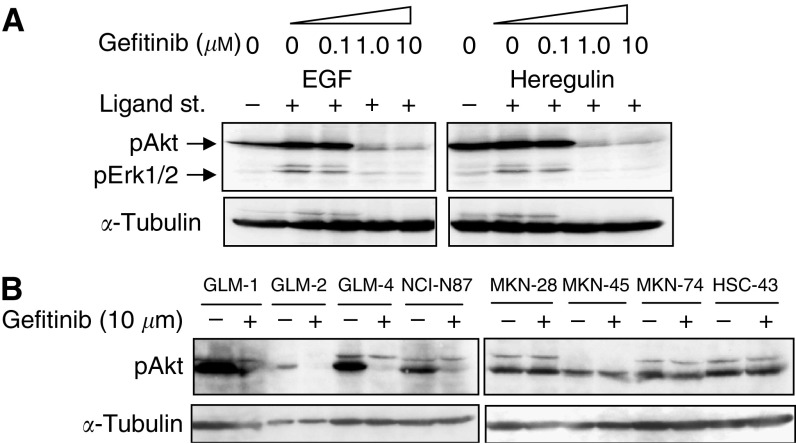
Effects of gefitinib on MAPK and PI3K/Akt signalling pathways in HER2-overexpressing cancer cell lines. (**A**) GLM-1 cells were cultured in serum-free medium for 24 h and exposed to gefitinib at increasing concentrations for 2 h at 37°C. Cells were then incubated with either EGF or heregulin (10 or 50 ng ml^−1^) for 10 min, lysed at 4°C in lysis buffer and subjected to SDS–PAGE, transferred to membrane. Immunoblot analyses were performed with specific antibodies against the phosphorylated Akt and Erk1/2. (**B**) Gastric cancer cell lines with HER2 overexpression (left) and low expression (right) were cultured in serum-free medium for 24 h and then treated with either gefitinib (1 *μ*M) or vehicle for 2 h. Phosphorylation of Akt was analysed by Western blot. Immunoblot with *α*-tubulin antibody was performed to evaluate protein loading.

HER2-overexpressing cell lines were supposed to upregulate the antiapoptotic PI3K/Akt pathway to achieve their growth and survival. To further clarify this point, we next examined the phosphorylation of Akt and Erk1/2 in HER2-overexpressing cells by Western blotting. EGF induced phosphorylation of Akt slightly, and Erk1/2 significantly, in GLM-1 cells, whereas heregulin induced significant phosphorylation only in Erk1/2. Such a ligand-induced phosphorylation was effectively inhibited by gefitinib at a concentration of 1.0 *μ*M (Figure 4A). Similar results were obtained from other HER2-overexpressing cell lines (GLM-2 and GLM-4). Furthermore, Akt, not Erk1/2, was constitutively phosphorylated in the absence of ligand stimulation. This constitutive phosphorylation of Akt was observed in both HER2-overexpressing and low-expressing cancer cell lines, but significant inhibition of the phosphorylation by gefitinib was observed only in HER2-overexpressing cell lines (Figure 4B). Expression of total PTEN, as well as phosphorylated PTEN, an antagonist of PI3K function and negatively regulates Akt activities, was not different between HER2-overexpressing and low-expressing cell lines (Figure 1B), indicating that PTEN is not involved in the constitutive activation of PI3K/Akt pathway in HER2-overexpressing cells.

### Effects of trastuzumab on the growth of HER2-overexpressing cells both *in vitro* and *in vivo*

**Figure 5:**
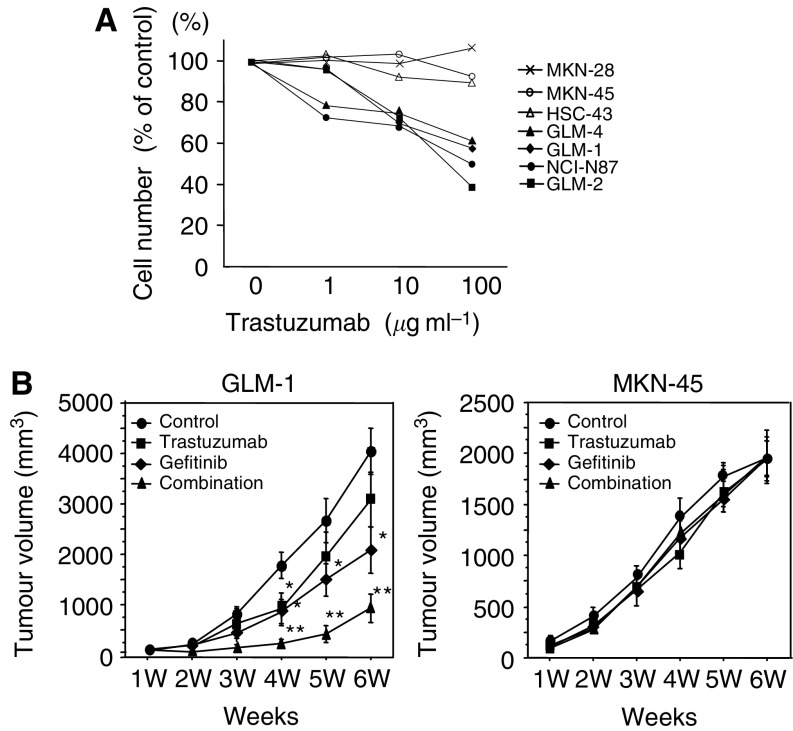
Growth inhibition by trastuzumab in HER2-overexpressing cells both *in vitro* and *in vivo*. (**A**) Growth inhibition by trastuzumab in various gastric cancer cell lines. Cells were plated in 24-well dish and treated twice with increasing doses of trastuzumab, followed by cell counting after a 4-day culture. (**B**) Inhibitory effects of gefitinib, trastuzumab and combination therapy on the growth of HER2-overexpressing (GLM-1) and low-expressing tumour (MKN-45) xenografts in nude mice. Tumour cells were injected subcutaneously into nude mice (*n*=5∼6) and gefitinib (150 mg per kg per day, oral administration, five times per week for 6 weeks), trastuzumab (20 mg per kg per day, intraperitoneal injection, twice a week for 6 weeks) and the combination was administered. ^*^P<0.01, ^**^P<0.001 (*vs* control). Bars=s.e.

Although growth inhibition of HER2-overexpressing cancer cell lines by trastuzumab (1–100 *μ*g ml^−1^) with maximally 50% of control was observed *in vitro*, it was less extensive than that by gefitinib (Figure 5A *vs* Figure 2A). Anti-tumour effect on the subcutaneously transplanted tumour (GLM-1) in nude mice was also observed by trastuzumab treatment, but was less extensive than by gefitinib. Furthermore, trastuzumab treatment in combination with gefitinib enhanced the antitumour effect of gefitinib additively. In contrast, such inhibitory effect of trastuzumab was not observed in the xenograft of the HER2 low-expressing cancer cell line (MKN-45) (Figure 5B).

### Isolation of gefitinib-resistant cell line, GLM-1R, from GLM-1

**Figure 6:**
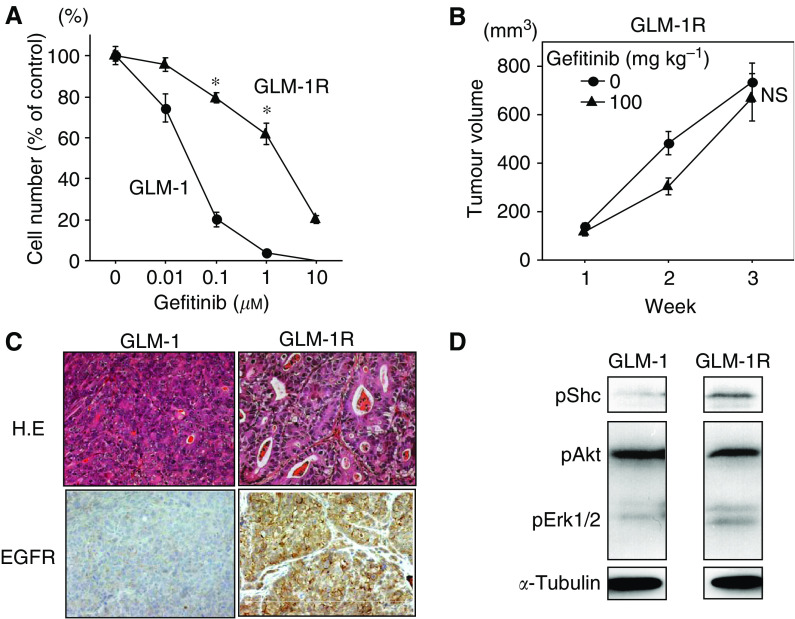
Growth characteristics and EGFR signalling of gefitinib-resistant GLM-1 cells (GLM-1R). (**A**) Comparison of growth inhibition by gefitinib *in vitro* between GLM-1 (•) and GLM-1R (▴) cells. ^*^P<0.01. (**B**) Effect of gefitinib on the growth of subcutaneous GLM-1R tumour xenograft in nude mice. Gefitinib was orally administered at a dose of 0 (•) and 100 mg per kg per day (▴), five times per week for 3 weeks. NS, not significant (*P*=0.69 *vs* control). (**C**) Comparison of histology and EGFR expression between GLM-1 and GLM-1R tumour xenograft in nude mice by immunohistochemistry. Cell surface EGFR expression was increased in GLM-1R cells. (**D**) Comparison of phosphorylation of Shc, Akt and Erk1/2 between GLM-1 and GLM-1R cells. Phosphorylation of Shc and Erk1/2 was increased in GLM-1R cells. Bars=s.e.

GLM-1 tumour xenograft in nude mice was not completely abolished by prolonged gefitinib treatment. To investigate such a acquired resistance of a gastric cancer cell line to gefitinib, we isolated the gefitinib-resistant cell line, GLM-1R, from parental GLM-1 cells by four cycles of selection of GLM-1 cell under exposure to gefitinib (1.0 *μ*M) for 2 weeks *in vitro*. GLM-1R cells were found to have a 65-fold higher IC_50_ for gefitinib (IC_50_=1.82 *vs* 0.028 *μ*M) (Figure 6A) and showed a significantly lower apoptosis induction by gefitinib than the parental cell line (data not shown). In addition, the growth inhibition of GLM-1R tumour xenograft in nude mice by gefitinib was no longer significant (Figure 6B).

### EGFR expression and downstream signalling in GLM-1R cells

GLM-1R tumour xenografts in nude mice exhibited more differentiated histology with well-defined tubular structures and stronger membranous staining of EGFR than parental GLM-1 tumour (Figure 6C), whereas HER2 expression remained unchanged between the two. Flow cytometric analysis confirmed that EGFR expression was significantly increased in GLM-1R cells as compared with parental GLM-1 cells (data not shown). Western blot analysis revealed that phosphorylation of Shc and Erk1/2, but not Akt, increased in GLM-1R cells in the absence of ligand stimulation as compared with parental GLM-1 cells (Figure 6D), indicating moderate, but constitutive activation of EGFR-MAPK signalling pathway in GLM-1R cells. Furthermore, a 10-fold higher concentration of gefitinib was needed for effective inhibition of phosphorylation of Akt in GLM-1 cells when EGF ligand stimulation increased from 10 to 50 ng ml^−1^ (data not shown), suggesting that the acquired resistance of GLM-1R cells to gefitinib may be due to the autocrine activation of the EGFR-MAPK pathway in compensation for PI3K/Akt pathway.

### Expression of EGFR, HER2 and pAKT in liver metastases from gastric cancer patients

To strengthen the HER2-driven constitutive activation of PI3K/Akt pathway in HER2-overexpressing cells *in vitro*, we examined the expression of phosphorylated Akt in the archived clinical specimens of liver metastasis from 20 gastric cancer patients, immunohistochemically. As shown in Table 1, HER2 overexpression was correlated with expression of phosphorylated Akt with marginal significance whereas EGFR overexpression was not correlated.

## DISCUSSION

In the present study, we found overexpression of HER2 in gastric cancer liver metastasis at a significantly higher incidence than primary tumour in the stomach. We newly established three HER2-overexpressing gastric cancer cell lines (GLM-1, GLM-2 and GLM-4) from such liver metastasis of Japanese patients (Nakanishi *et al*, 2005), and demonstrated for the first time that they were highly sensitive to gefitinib rather than trastuzumab. In fact, gefitinib induced remarkable apoptosis in these HER2-overexpressing cells, that has never been observed in conventional gastric cancer cell lines without HER2 overexpression. We then examined another HER2-overexpressing intestinal-type gastric cancer cell line, NCI-N87, derived from liver metastasis of a Caucasian patient (Park *et al*, 1990), and confirmed the high gefitinib sensitivity such as that found in the GLM series. Interestingly, most of these HER2-overexpressing cell lines express CXCR4, which is reportedly associated with haematogenous metastasis in breast, colon and prostate cancer patients (Muller *et al*, 2001; Brand *et al*, 2005), and is known to be regulated by HER2 through the PI3K/Akt pathway (Li *et al*, 2004). These clinicopathological findings strongly suggest that HER2-overexpressing gastric cancer liver metastases are a unique clinical entity sharing the same characteristics, for example, morphology (intestinal type), metastatic potential (CXCR4 expression) and gefitinib sensitivity, and therefore, may be a good therapeutic target for gefitinib. According to the past international Phase II randomised clinical trial consisting of 75 stage IV gastric cancer patients (32 Japanese and 43 non-Japanese from Europe, Australia and Singapore), the result was quite pessimistic, showing the absence of a complete response (0%), a very low partial response rate (1.4%), and a low rate of stable disease (17%); the overall therapeutic efficacy of gefitinib was quite low (18.3%) (Doi *et al*, 2003; Rojo *et al*, 2003). In this trial, however, no patient selection based on the prediction of gefitinib sensitivity was performed. Therefore, it is reasonable to assume that, if HER2-overexpressing gastric cancer patients with liver metastasis are prospectively selected, the response rate of gastric cancer patients owing to gefitinib may be significantly increased.

The important issue in this study is the demonstration of the possible mechanism and biological significance of constitutive activation of PI3K/Akt pathway in the HER2-overexpressing gastric cancer cell lines. Several things might explain the mechanism of constitutive activation of PI3K/Akt pathway. PTEN, a phosphatase converting PIP3 to inactive PIP2 antagonises PI3K function and negatively regulates Akt activities. Thus, loss of PTEN function owing to mutations, haploinsufficiency from LOH and hypermethylation, which are known to be present in 30–40% of gastric cancers (Kang *et al*, 2002; Oki *et al*, 2005), can lead to the constitutive activation of PI3K/Akt pathway. In this study, however, PTEN protein expression in both total and phosphorylated form was not downregulated as compared with HER2 low expressing gastric cancer cell lines, suggesting that PTEN is not involved in the constitutive activation of PI3K/Akt pathway in HER2-overexpressing cells. On the other hand, a recent study on the crystal structure of HER2 protein demonstrated that HER2 has a fixed conformation that resembles a ligand-activated state and can interact with other HER receptors such as EGFR and HER3 in the absence of direct ligand binding (Cho *et al*, 2003). Alimandi *et al* (1995)Previously reported the increased recruitment of PI3K by the formation of HER2/HER3 heterodimer in a co-transfection experiment with HER2 and HER3. Furthermore, Arteaga *et al* demonstrated HER2/HER3 heterodimer formation, the association of p85 subunit of PI3K with HER3 and subsequent PI3K/Akt activation in HER2-overexpressing breast cancer cell lines (Moulder *et al*, 2001; Anido *et al*, 2003). Based on these findings, it is reasonable to speculate that overexpression of HER2, together with relatively high expression of HER3 in GLM-1, GLM-2 and NCI-N87 cells, can promote HER2/HER3 heterodimer formation, followed by association of HER3 with PI3K, which in turn induce the constitutive activation of the downstream PI3K/Akt pathway. As for biological significance of this activation, we found that PI3K inhibitor (LY294002), not MEK1/2 inhibitor (U0126), abolished phosphorylation of Akt and induced strong apoptosis in HER2-overexpressing cells, whereas PI3K inhibitor reduced phosphorylation of Akt without inducing apoptosis in HER2 low-expressing cells. This suggests that HER2-overexpressing cell lines strongly depend for their survival on the constitutively activated PI3K/Akt pathway as a predominant downstream signalling pathway, whereas HER2 low-expressing cells use multiple signalling pathways other than the PI3K/Akt pathway for their survival.

Although HER2-overexpressing gastric cancer cell lines thus far proved to addict to the self-activated PI3K/Akt pathway, it is still somewhat puzzling that gefitinib is a tyrosine kinase inhibitor specific for EGFR, yet exhibits a marked antitumour effect against HER2-, but not EGFR- overexpressing gastric cancer cells. Anido *et al* (2003) reported the gefitinib-sensitive HER2- overexpressing breast cancer cell lines and proposed the hypothesis that gefitinib abolishes the formation of HER2/HER3 heterodimers by promoting sequestration of HER2 and HER3 into inactive (unphosphorylated) EGFR/HER2 and EGFR/HER3 heterodimer. In the present study, we found that gefitinib can selectively abolish constitutive phosphorylation of Akt only in HER2-overexpressing cells, although HER2 low-expressing cells also show constitutive activation of the PI3K/Akt pathway, suggesting that gefitinib can selectively block constitutively activated PI3K/Akt pathway only in the case simply driven by HER2 overexpression along with relatively high expression of HER3, which permit to form HER2/HER3 heterodimer. This finding is consistent with the reports by Anido *et al*, but further study is warranted to prove this point. On the other hand, we found that three of four HER2-overexpressing, gefitinib-sensitive gastric cancer cell lines have HER2 gene amplification. Therefore, an alternative explanation for the high gefitinib sensitivity, specific for HER2-overexpressing gastric cancer cell lines, is that these lines have other concomitant genetic changes such as somatic mutations within the ATP-binding pocket of the tyrosine kinase domain of EGFR (or HER2), which is known to confer susceptibility to gefitinib in lung adenocarcinoma patients as reported previously (Sordella *et al*, 2004; Stephens *et al*, 2004; Mitsudomi *et al*, 2005). In the present study, however, we could not detect any mutations in EGFR and HER2 tyrosine kinase domain of GLM-1 and GLM-2 cells, such as small in-frame deletions and missense substitutions, which might rule out the possibility of mutations as the cause of gefitinib sensitivity.

Another interesting finding in this study is the acquired resistance of HER2-overexpressing gastric cancer cell lines to gefitinib. Gefitinib-resistant GLM-1R cells exhibited increased EGFR expression and more differentiated morphology as compared with parental GLM-1 cells. Shc and Erk1/2, respective upstream and downstream signalling molecules of the MAPK pathway, were also upregulated in GLM-1R cells. Furthermore, inhibition of Akt phosphorylation in parental GLM-1 cells in response to gefitinib treatment attenuated when Ras/MAPK pathway was strongly stimulatied by a higher EGF concentration (50 *vs* 10 ng ml^−1^). These findings suggest that the EGFR-Ras-MAPK pathway is constitutively (but moderately) activated in compensation for the blockade of the HER2-PI3K-Akt pathway in GLM-1R cells, resulting in persistent growth in the presence of gefitinib and therefore acquired resistance. Consistent with our finding, Jones *et al* reported that IGF-1R signalling pathway serves as a bypass for the EGFR-MAPK pathway, and is involved in the acquired resistance to gefitinib in a human breast cancer cell model (Jones *et al*, 2004). Second mutations of the tyrosine kinase domain in EGFR, and gain-of-function mutations in downstream signalling molecules of Ras/MAPK and PI3K/Akt pathways, including PTEN, are other potential mechanisms of aquired resistance to gefitinib (Kobayashi *et al*, 2005; Koizumi *et al*, 2005; Shih *et al*, 2005). In the present study, at least mutations in EGFR and HER2, and loss of PTEN function were not observed in GLM-1R cells, suggesting that the compensation for PI3K/Akt pathway by upregulation of Ras/MAPK pathway is the major cause of gefitinib resistance in GLM-1R cells. We found that the residual tumour xenograft in nude mice 1–2 month after gefitinib treatment also showed upregulation of EGFR. Therefore, GLM-1 cells can acquire resistance to gefitinib after prolonged treatment *in vivo*. These findings suggest the potential importance of bi- or multi-directional inhibition of the PI3K/Akt, Ras/MAPK and other pathways for more effective treatment of HER2-overexpressing gastric cancer cell lines. Now, we are also investigating the antitumour activity of a combination of both inhibitors for PI3K and MAPK pathway against GLM-1R cells.

In conclusion, we identified for the first time a new clinical entity – a subset of gastric cancers showing high susceptibility to gefitinib and liver metastasis. Our immunohistochemical finding obtained from archived clinical specimens, that Akt pathway tended to be activated (phosphorylated) in the liver metastasis in HER2-overexpressing gastric cancer patients, while still preliminary, suggests that HER2 overexpression, together with constitutive activation of the PI3K/Akt pathway, may be useful predictive markers for gefitinib sensitivity in gastric cancers. Therefore, HER2-overexpressing gastric cancer with liver metastasis would be a potential target for molecular therapy with gefitinib and related compounds.

## Figures and Tables

**Table 1:** Immunohistochemical analysis of EGFR, HER2 and phosphorylated Akt (pAkt) expression in metastatic liver tumours from 20 gastric cancer patients

		**pAkt**		
**Staining**	**No. of patients examined**	**(−)**	**(+)**	***P*-value**
*HER2*				
(−)	7	6	1	0.057^a^
(+)	13	4	9	
				
*EGFR*				
(−)	16	8	8	>0.999
(+)	4	2	2	

a *χ*^2^ test, *P*-value=0.0191, Fisher's exact test, *P*-value=0.0573.
